# Therapeutic Potential of Plant- and Marine-Derived Bioactive Compounds in Prostate Cancer: Mechanistic Insights and Translational Applications

**DOI:** 10.3390/ph18030286

**Published:** 2025-02-20

**Authors:** Arif Jamal Siddiqui, Mohd Adnan, Juhi Saxena, Mohammad Jahoor Alam, Abdelmushin Abdelgadir, Riadh Badraoui, Ritu Singh

**Affiliations:** 1 Department of Biology, College of Science, University of Ha’il, Ha’il P.O. Box 2440, Saudi Arabia; drmohdadnan@gmail.com (M.A.); jahooralam@gmail.com (M.J.A.); abdelmuhsin@yahoo.com (A.A.); badraouir@yahoo.fr (R.B.); 2 Department of Biotechnology, Parul Institute of Technology, Parul University, Vadodara 391760, Gujarat, India; jina.saxena@gmail.com; 3 Department of Environmental Science, School of Earth Sciences, Central University of Rajasthan, Ajmer 305817, Rajasthan, India

**Keywords:** prostate cancer, natural product, marine compounds, anticancer properties, plant-based compounds

## Abstract

It is widely recognized that prostate cancer is a multifaceted illness that is the second most common cause of cancer-related fatalities among males. Natural sources from both plants and marine organisms have long been used in treating various diseases and in the discovery of new pharmaceutical compounds. Medicinal plants, in particular, provide bioactive substances like alkaloids, phenolic compounds, terpenes, and steroids. In addition, marine natural products play a crucial role in the search for novel cancer treatments. A substantial number of anticancer drugs have been derived from natural sources, including plants, marine organisms, and microorganisms. In fact, over the past 60 years, 80% of new chemical entities have originated from natural sources, which are generally considered safer than synthetic compounds. This review seeks to emphasize the role of phytochemical compounds derived from both plant and marine sources in prostate cancer, highlighting their potential therapeutic impact. It is also intended to support global researchers working on the identification of natural-based treatments for prostate cancer.

## 1. Introduction

### 1.1. Prostate Cancer

Over the last century, the escalation of chronic diseases such as malignancies has presented a significant challenge to people’s health systems in developing countries. It is a fact that cancer is a significant health problem in several countries, and it is characterized by increasing prevalence, mortality rates, and significant treatment costs across all genders and age groups [1]. It is widely recognized that cancer causes a significant amount of complication and represents the disease with the second-highest incidence worldwide [1,2,3]. Cancer arises from an unregulated proliferation of cells that occurs in multiple tissues and can subsequently invade neighboring and distant tissues [4]. Despite significant advances in the field of cancer biology, cancer remains a leading cause of death. In addition, individuals who survive cancer often face long-term problems, including physical, cognitive, and psychological challenges, as well as side effects of therapy [4,5]. Cancer is becoming more prevalent all around the world, which is a cause for significant concern. Several risk factors associated with this upward trend have been identified, with lifestyle changes playing a major role. In oncology research, several cancers have been recognized as commonly diagnosed, including prostate, breast, stomach, lung/bronchus, liver, and colorectal cancers [6]. In 2018, prostate cancer had the highest incidence rate among cancers diagnosed in the United States of America, Oceania, and Europe [7]. A considerable focus has been placed on prostate cancer over the last decade due to the concerning prevalence of affected individuals and increased mortality rates [8]. After skin cancer, prostate cancer is the second most lethal cancer among males. Furthermore, it is noteworthy that this particular cancer is generally considered to be the most common cancer in the male population and has a significant mortality rate. In 2022, over 1.5 million cases of prostate cancer were identified [9]. When comparing the data from 2012 to today, it is clear that there has been a significant increase of around 45% in the incidence rate and a corresponding increase of 19% in the death rate [10,11,12]. Based on data from the American Cancer Society, it is estimated that about one in nine men will be diagnosed with cancer during their lifetime, with prostate cancer being the cause of death in roughly one in forty-one men [13]. The prostate gland is an organ situated posterior to the bladder and comprises epithelial cells arranged inside a fibromuscular stromal network [14]. Despite the challenges in definitively determining the etiologic variables that link the development of prostate cancer to its prevalence, numerous studies have consistently identified common risk factors associated with the disease, including dietary habits, race, physical activity, and age [15,16,17,18]. The incidence of prostate cancer is mainly influenced by age factors, as the likelihood of developing this disease tends to increase with age [18]. Attard et al. (2010) identified family history, namely the presence of prostate cancer in a first-degree relative, as the most important risk factor, along with race and age [19]. Pandey et al. have stated that the etiology of prostate cancer is influenced by either genetic or somatic mutations and accounts for a maximum of 10% of cases [20]. The remaining 90% of prostate cancer cases are attributed to epigenetic changes, particularly those related to lifestyle factors. Nevertheless, it is known that inflammation is a progression associated with high-risk elements of cancer. To gain a comprehensive understanding of the substantial impact of inflammation on cancer, it is crucial to address the pathological and physiological mechanisms associated with inflammation [21]. The timely identification of this cancer, similar to other types of cancer, holds significance in terms of improving treatment outcomes, mortality prevention, and reducing morbidity rates. Consequently, numerous studies have been undertaken to assess the potential danger of prostate cancer by examining various initial symptoms and signs [22,23]. Several studies have focused on LUTSs (lower urinary tract symptoms) such as urinary retention, nycturia, and hesitancy. However, the consensus among these studies is that there are no highly predictive signs and symptoms of prostate cancer [24]. Researchers state that PSA (prostate-specific antigen) screening and DRE (digital rectal examination) are highly recommended for all prostate cancer patients with lower urinary tract symptoms (LUTSs), highlighting the crucial role of primary care physicians and general practitioners in the detection of prostate cancer in this population. Referral to a urologist for additional assessment and to differentiate benign prostatic hyperplasia from prostate cancer is advised if anomalies are found [10]. In 1987, PSA testing was initially implemented to evaluate the efficacy of treatments for prostate cancer. Later on, though, it also used as a prostate cancer screening tool [25,26]. Following the pervasive utilization of PSA as a screening test, a significant increase in the number of diagnoses cases was observed between 1989 and 1992. From 1995 onwards, this upward trend persisted, albeit with a slight decline in slope until 2001. Subsequently, incidence rates fluctuated yearly, suggesting differences in screening practices [10]. Since the implementation of prostate cancer screening using PSA in 1991, there has been a noticeable decrease in mortality rates. This fall might be attributed to the early identification of the disease and the subsequent appropriate treatment and care provided to patients [27]. Testing for prostate-specific antigen (PSA) requires a level of at least 4.0 ng/mL. Well-defined studies show that the negative predictive value of PSA for the primary identification or diagnosis of prostate cancer in men with an average age between 65 and 70 years is 89% when this cutoff number is used [28]. Consequently, prostate biopsies, magnetic resonance imaging (MRI), and whole-body bone scans should be considered for patients whose PSA levels in two independent examinations are more than 4 ng/mL [10]. It is important to remember, however, that prostate-specific antigen (PSA) does not necessarily indicate the presence of prostate cancer. Furthermore, it is essential to keep in mind that the vast majority of prostate tumors do not constitute a substantial risk if they are both discovered and treated in a timely manner. As a result, there is a contentious discussion going on right now regarding the efficacy of PSA screening in the early diagnosis of prostate cancer [29,30]. Consequently, scholars have proposed alternative biomarkers, including free PSA, prostate-specific membrane antigen, prostate cancer antigen 3, human kallikerin 2, and others, to enhance the accuracy of prostate cancer diagnosis and mitigate the issues of excessive diagnosis and treatment [31]. However, it is widely agreed upon by the public that by considering patient risk factors, medical practitioners can effectively identify high-risk individuals and prioritize their care, thereby minimizing the likelihood of overlooking clinically significant cancer cases and reducing the incidence of unnecessary treatment and diagnosis [32]. The presence of heterogeneity in prostate cancer requires the assessment of risk variables as a crucial step in predicting disease behavior [33]. Numerous analyses of epidemiological research have steadily highlighted the perception that natural dietary substances exhibit chemopreventive properties and have the potential to prevent various types of malignancies, such as prostate cancer, breast cancer and other cancers [34,35,36]. Nowadays, plant-derived phytochemicals are receiving more attention due to their potential to combat the spread of neoplastic diseases due to their efficiency, as well as their low number of drawbacks [3,37]. Additionally, several plant-sourced natural compounds of medical significance, such as taxol, atropine, morphine, digitoxin, and theobromine, are currently manufactured and commercialized. Thus, molecules from nature have been recommended for the sustainable use of medicinal plants and marine sources [38].

Nevertheless, there is a lack of consistency in the research that has been conducted on the optimal plant-based diet, as well as on the associated nutrients and phytochemicals. Furthermore, the effects that plant- and marine-derived bioactive compounds have on the health of the prostate are not well documented and need to be further studied. This review aims to identify naturally occurring active compounds derived from plants and marine sources whose effects, particularly on prostate cancer, have not been sufficiently addressed or fully explored, including their detailed mechanistic insights, clinical findings, and translated applications. This review also particularly focuses on the integration of nanotechnological formulations and combinatorial therapies targeting prostate cancer.

### 1.2. Epidemiology

In terms of incidence and cancer-related mortality in men, prostate cancer ranks second in the world and fifth in men. In 2018, 0.359 million people died, and 1.276 million cases were reported. After lung, breast, and colon cancer, it is the fourth most common cancer in terms of incidence [7,39]. Furthermore, according to Bray et al. (2018), it is the seventh leading cause of death in both men and women [40]. By 2040, there are predicted to be 2.2938 million cases of prostate cancer (PCa) worldwide if current trends continue. Prostate cancer (PCa) is the most common disease worldwide, accounting for over 50% of cases diagnosed (105 of 185). In terms of the mortality rate from prostate cancer, the highest rate is seen in Southern Africa (26.4%), followed by the Caribbean (25.4%) and Central Africa (22.4%). Prostate cancer, often known as PCa, is the most prevalent form of cancer within the male population in both the United States and the United Kingdom [40,41].

## 2. Terrestrial Plants as Sources of Natural Products for Prostate Cancer Therapy

**Figure 1:**
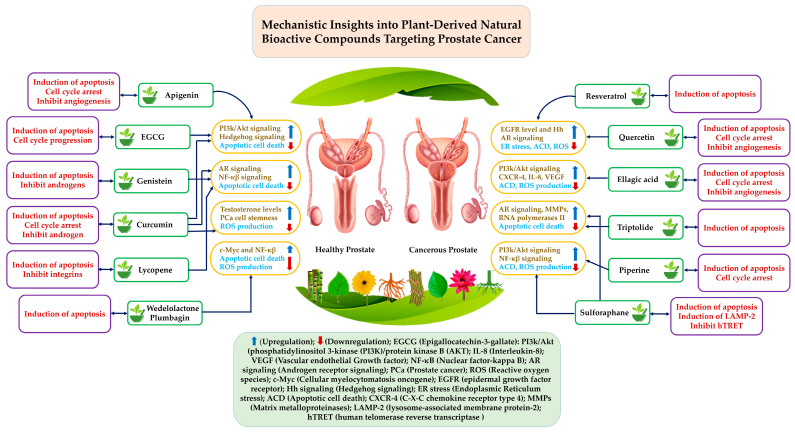
Presentation of natural bioactive compounds and their mechanism of action against prostate cancer.

Traditional medicinal plants have been used to treat and cure a wide range of disorders, which has led to an increase in the utilization of medicinal plants in the hunt for novel medications that are derived from nature. The discovery that plant extracts have the potential to treat human ailments is frequently the impetus behind the creation of new pharmaceuticals. Natural bioactive compounds, such as secondary metabolites, could be obtained from plants [42]. According to Chaachouay (2024), these secondary metabolites are present in every component of a plant, including seeds, flowers, leaves, and bark [43]. Even the seeds themselves contain secondary metabolites. Bodeker pointed out that secondary metabolites are important and crucial for the human consumption of plants, even though there is a lack of knowledge regarding the fundamental processes that are responsible for the processing of secondary metabolites in plants [44]. It is well known that the use of natural-based herbal medicine has very few side effects and toxicity properties compared to conventional or synthetic chemotherapy drugs [45]. Moreover, presently, important herbal medicines are significantly and progressively more used in cancer treatment and represent a rich source of novel and bioactive chemical compounds that can be used in the production of chemotherapy drugs. From this point of view, the following section provides a detailed description of the plant extracts and the associated bioactive components that can combat prostate cancer, as shown in Table 1 and Figure 1. In addition, a particular focus will be placed on clinical studies that provide evidence of phytochemicals derived from plants that have the ability to combat prostate cancer.

### 2.1. Apigenin

A flavonoid known as apigenin can be found in the Anthemis species, which is ingested by humans. The family Asteraceae and other plants possess a wide range of medicinal qualities, such as anticancer, neuroprotective, antioxidant, antiviral, anti-inflammatory, and antimetastatic activities. It is essential to take into consideration the fact that apigenin affects normal cells with only a modest amount of toxicity. It has shown an anticancer effect against a wide range of malignancies, including prostate cancer (PCa), in addition to other types of cancer. It exerts this effect by inhibiting the development of SPOCK1, an acronym for SPARC (Osteonectin), Cwcv, and Kazal Like Domains Proteoglycan 1. As a result, it decreases the expression of mesenchymal markers in several prostate cancer cells. According to Chien et al.’s 2019 study, apigenin inhibits the growth, proliferation, invasion, and spread of PC3-induced xenograft tumors thanks to its influence [75]. In a study carried out by Kaur and colleagues (2008), it was discovered that it inhibited the activity of p-Akt (S473), p-GSK3, and p-BAD, in addition to inhibiting the production of IGFR and its autophosphorylation [47]. In addition, it stimulated the process of apoptosis, which results in the death of cells in both laboratory tests and in living beings. By targeting the PI3K/Akt/FOXO signaling pathway, apigenin decreased the growth and proliferation of prostate cancer tumors. Based on the study’s findings, apigenin was found to cause a cell cycle arrest in the G0/G1 phase [76]. Furthermore, it was found to be effective in inhibiting the formation of cMyc and catenin signals while simultaneously boosting the concentrations of BIM and p27/Kip1 [76,77]. By regulating the cell cycle, apigenin is able to limit the proliferation of cells that are associated with prostate cancer. Kip1/p27, Waf1/p21, INK4a/p16, and INK4c/p18 were all upregulated, whereas cyclins D1, D2, E, and Cdk2, Cdk4, and Cdk6 were downregulated. This allowed for the effective completion of the task. Additionally, apigenin phosphorylated the retinoblastoma protein at S780. In both 22Rv1 and PC3 cell-induced xenograft tumors, it decreased the contact between cyclin E and Cdk2 while simultaneously increasing the interaction between cyclin D1 and Waf1/p21 and Kip1/p27. In a study conducted by Shukla and Gupta (2006), it was proven that apigenin can stabilize p53 function by phosphorylating serine 15 in tumors produced in 22Rv1 cells [78]. Additionally, it triggered reactive oxygen species (ROS) formation and p53-dependent apoptosis in experiments conducted in vitro and in vivo. Furthermore, it boosted the downregulation of MDM2 protein through p14ARF while simultaneously blocking the activity of NF-B/p65 [77]. The bioactive compound apigenin has the capability to successfully obstruct the proliferation of cells in laboratory models such as in vitro and in vivo experiments. This was achieved by modifying the IGF/IGF-IR signaling pathway, more specifically by increasing IGFBP-3 and p27/Kip1 levels and by decreasing the activity of p-Akt, p-GSK3, and cyclin D1 [47]. The activity of class I HDACs, particularly HDAC1 and HDAC, is suppressed by apigenin, leading to cell cycle arrest and apoptosis and thus preventing tumor formation. According to the results of research by Pandey and colleagues (2012), this leads to an increase in the acetylation of H3 and H4, as well as the excessive acetylation of H3, especially at the p21/Waf1 promoter [79]. In addition, it affects the ratio of Bax to Bcl-2 and causes an increase in the expression of p21 and Waf1 in various tumor tissues. One study showed that apigenin was effectively able to inhibit the development of new blood vessels in tumor tissue by downregulating the expression of HIF1 and VEGF [80].

### 2.2. Epigallocatechin-3-Gallate

Green tea made from *Camellia sinensis* contains a polyphenolic compound called epigallocatechin-3-gallate (EGCG). This molecule is important because it is found in plants [81]. According to Chu et al. (2017), EGCG contains a wide range of pharmacological activities, including antioxidant, anticancer, antidiabetic, cardioprotective, and neuroprotective effects [82]. According to Hagen et al. (2013), activation of caspase 9 is the mechanism by which EGCG results in the death of PC3 cells when administered alone and in combination with cisplatin [83]. According to Harper et al. (2007), the chemical EGCG is responsible for the regression of prostate cancer in TRAMP mice. This is achieved by reducing the effects of the androgen receptor (AR), insulin-like growth factor 1 (IGF-1), and its receptor, insulin-like growth factor receptor 1 (IGF-R1) [84]. Previous studies demonstrated that EGCG has a tendency to generate apoptosis in both androgen-dependent (LNCaP) and androgen-independent (DU-145) cells via a mechanism that does not directly involve the p53 protein [85]. Furthermore, regulating the activities of two kinase inhibitors, namely, cyclin-dependent kinases and cyclin kinase inhibitors, showed that EGCG was most responsible for LNCaP and DU-145 cells failing to progress through the cell cycle and even dying [85]. In 2003, Hastak and colleagues conducted a study in which they discovered that EGCG has the capability to initiate apoptosis progression in human prostate cancer cell lines by activating p53 and inhibiting the NF-kB pathway. Moreover, all these mechanisms happen by increasing the proportion of Bax to Bcl-2. However, in response to R1881, EGCG inhibits the migration of AR to the nucleus and reduces the amount of protein contained therein [86]. Furthermore, in a PCa xenograft model, EGCG decreased the level of miRNA-21, which is regulated by androgens, and increased the level of miRNA-330, which acts as a regulator of tumor growth (Siddiqui et al., 2011). Both effects were observed in a PCa treatment model [87]. A study conducted by Stearns et al. (2010) found that the combination of two bioactive compounds, doxorubicin and EGCG, could effectively reduce the development of tumors made by metastatic PC-3M cells in CB17-SCID mice [88]. According to the results of research conducted in 2014 by Wang et al., the combination of two bioactive molecules, quercetin and green tea rich in EGCG, was able to significantly decrease the progression of tumors generated by androgen-sensitive LAPC-4 cells in the SCID mouse model [89]. Thus, by reducing the levels of inflammation, collagen deposits, oxidative stress, and angiogenic growth factors, EGCG is able to alleviate the symptoms of benign prostatic hyperplasia and fibrosis, which happen as a consequence of an increase in the level of testosterone in rats. In addition, it can suppress the overproduction of HIF-1, TGF-RI, p-Smad3, TGF-1, ER-, and AR and increase the levels of ER- and miR-133a/b, as already mentioned by Zhou et al. in their 2018a study [90]. By activating the downregulation of Akt1 through the mediation of miR-520a-3p, the chemical EGCG was able to trigger programmed cell death in prostate cancer cells, as well as in tissue samples from patients [91]. Vasculogenic mimicry, also known as VM, is a process used in advanced prostate cancer (PCa) to create vascular-like networks that are essential for tumor growth and aggressiveness. Furthermore, the use of EGCG therapy in PC3 cells can successfully block vascular membrane (VM) adhesion by targeting several proteins involved in this process. These proteins include vimentin, vascular endothelial cadherin, Akt, Twist, N-cadherin, and p-Akt [92]. According to research by Marchetti et al. from 2020, EGCG is responsible for the death of PCa cells because it causes a breakdown in the calcium homeostasis of the cells during the apoptotic process [93].

### 2.3. Genistein

Genistein is an isoflavone that can be obtained from legumes of the Leguminosae family. Some examples of legumes that contain genistein are chickpeas (Cicer arietinum), broad beans (Vicia faba), and soybeans (Glycine max). According to Ghosh et al. (2016), the anti-inflammatory effects of genistein are well documented [94]. There have been many studies that have shown that genistein may destroy cells (cytotoxic potential) in a variety of cancers, including prostate cancer (PCa) [95,96]. This is effectively achieved by influencing the activity of several signaling pathways, particularly AR, PI3K/Akt, NF-B, Hh, and Wnt. In a study published in 2020, Fontana and colleagues showed that genistein could efficiently limit the synthesis of several cancer-causing microRNAs, reduce the spread of cancer to the bone, and suppress the stem cell-like properties present in prostate cancer [46]. Research conducted by Shafiee and colleagues in 2020 discovered that it could successfully inhibit the development and proliferation of PC3 cells by inhibiting the activities of the p38MAPK and MMP2 proteins [97]. In addition, it activated caspase 3, which led to implementing a planned cell death mechanism. By producing ROS in PC3 and LNCaP cells, the combination of genistein and celecoxib delivered in nanoliposomes resulted in the death of cells through a process known as apoptosis. Significantly, this therapy caused no damage to normal fibroblasts. In addition to suppressing COX-2 synthesis and the function of Glut-1 transporters, the mechanism responsible for this selective effect involved a reduction in the levels of glutathione (GSH) and peroxiredoxin-6 (Prx-6), which are present in cells in the body. Tian and his colleagues collaborated to conduct this research in 2019 [98]. As shown by Song et al. (2020), the use of a nanoliposome formulation containing genistein and plumbagin resulted in the development of apoptotic cell death in PC-3 and LNCaP cell lines as well as in vivo in mice. This result was achieved by inhibiting the PI3K/Akt signaling pathway and the Glut-1 transporters [99].

The combination of genistein combined polysaccharide (GCP) inhibits the production of androgens within cells by decreasing testosterone levels by approximately three times and lowering the amount of PSA. A chemical was shown to be effective in inhibiting the production of enzymes that participate in the intracrine synthesis of AR. These enzymes include CYP17A, StAR, 17βHSD, SRB1, and 3βHSD. According to a study by Batra et al. from 2020, this inhibition caused apoptosis, which in turn led to a reduction in cell growth and proliferation that occurred in LNCaP and PC346C cells [100]. According to a study by Altaf et al. from 2019, genistein has the ability to enhance the effect of a moderate dose of radiation (20 mGy/h) on DU145 cells, ultimately leading to a more efficient induction of apoptotic cell death [101]. In another study conducted by Zhang (2019), genistein, a chemical found in soy products, was shown to inhibit the signaling pathways responsible for converting a less aggressive form of prostate cancer (PCa) into a more lethal and mobile phenotype [102,103]. Furthermore, consuming soy products containing genistein over a long period can potentially lead to adaptive changes in biomarkers related to cell motility in human PCa cells. These biomarkers include MEK4 and MMP2. It was found that pretreatment with genistein resulted in significant changes in DNA methylation and gene expression in people with prostate cancer, as opposed to people treated with a placebo [104,105]. This was observed during a clinical experiment conducted. According to Bilir et al. (2017), therapy with genistein resulted in a reduction in MYC activity and an improvement in PTEN function [106].

### 2.4. Curcumin

Curcumin, a polyphenol molecule derived from Curcuma longa, has been reported to have various pharmacological potentials, including antioxidant, anticancer, neuroprotective, and anti-inflammatory properties. According to many researchers, curcumin can exert its effects by selectively altering a variety of signaling pathways [107,108]. These signaling pathways include NF-B, Wnt/Catenin/TGF/MYC signaling, AR signaling, PI3k/Akt/mTOR signaling, and AP-1 signaling. Due to curcumin’s ability to influence several signaling pathways simultaneously, it should be no surprise that it is a potent inhibitor of prostate cancers [109]. By suppressing the activity of epidermal growth factor receptor (EGFR) tyrosine kinase, curcuminoid can induce apoptosis in PC3 and LNCaP cells [109]. By activating TNF and suppressing -catenin, NF-kB, and protein kinase D (PKD), curcumin induces apoptosis in DU145 cells [110,111]. This is achieved by decreasing the expression of Bcl2 and increasing the expression of caspase-3 and caspase-8. Khor et al. (2011) discovered that curcumin has the ability to reverse the hypomethylation of CpG islands at the NRF2 promoter in TRAMP cells and therefore increases the production of NRF2 and activates the cellular antioxidant system [112]. In 2011, Teiten et al. showed that it is effective in combating prostate cancer by blocking the Wnt/Catenin pathway in an androgen receptor-dependent manner while triggering cell cycle arrest during the G2 phase and autophagy [113]. There is evidence that curcumin inhibits glyoxalases, increases methylglyoxal levels, and decreases cellular glutathione (GSH) and ATP levels, all contributing to the destruction of prostate cancer [114]. Furthermore, a study conducted by Huang (2015) found that combining curcumin and tomatoes could successfully inhibit the growth of tumors in a PC3 xenograft model using the combination [115]. Curcumin was discovered to have the ability to prevent the formation of cancer-associated fibroblasts (CAFs). These are cells involved in tumor growth, invasion, metastases, and the epithelial–mesenchymal transition (EMT) in prostate cancer. According to the results of a study by Du et al. (2015), it has the ability to reduce the activation of CAFs caused by the production of ROS, interleukin-6 (IL-6), and CXC chemokine receptor-4 (CXCR-) expression [116]. Monoamine oxidase-A (MAO-A), mammalian target of rapamycin (mTOR), and hypoxia-inducible factor 1 alpha (HIF-1) are all included in the signaling pathway that is suppressed to achieve this result. Curcumin analogs were found to inhibit androgen receptor (AR) activity, which is induced by testosterone and DHT, according to a study that was conducted by Schmidt and Figg 2016 [117]. Moreover, according to the findings of a recent study that was carried out by Tanaudommongkon et al. in the year 2020, curcumin-loaded lipid nanoparticles (NPs) exhibited significant cytotoxicity in docetaxel-resistant DU145 and PC-3 cells [118]. The combination of curcumin and cabazitaxel encapsulated in lipid–polymer hybrid nanoparticles (aptamer-PLGA-PEG) has shown improved efficacy in drug delivery and the induction of cytotoxicity in prostate cancer cells in both in vitro and in vivo experiments [119]. According to the research of Eslami et al. from 2020, the co-injection of curcumin and metformin into PCa cells increased both spontaneous and planned cell death (apoptosis) [120].

### 2.5. Ellagic Acid

A polyphenolic compound known as ellagic acid is found in many fruits and vegetables, including Vaccinium species in the family Ericaceae and Rubus species in the family Rosaceae. Its numerous properties, such as anticancer and antioxidant properties, have earned it a good reputation. On the other hand, capazotaxel inhibits tubulin polymerization while promoting microtubule assembly. In castration-resistant PCa/22Rv1 cells, ellagic acid was found to be effective in preventing drug clearance, as Eskra et al. determined [121]. On the other hand, it did not affect the effectiveness of docetaxel in living organisms. In addition to modulating the Bcl2-to-Bax ratio and caspase-3 levels, it blocked the growth of tumors by causing caspase-3-mediated cell death in a TRAM cancer model. This was achieved by inhibiting tumor growth. Research conducted by Naiki-Ito and colleagues (2015) found that LNCaP cell proliferation was decreased [122]. Parallel to this decline were increased protein levels associated with the cell cycle. These proteins included cyclin E, cdk2, P27Kip, and p21Waf. Sprague Dawley rats also showed an observed decrease in the expression levels of Cdk1 and Cyclin D1. Ellagic acid co-administrated with luteolin and punicic acid could effectively reduce angiogenesis, metastasis, and tumor formation in allograft and xenograft tumors formed by PC-driven tumor cells [47]. Wang et al. (2014b) reported that ellagic acid could successfully reduce the activities of p-Akt/PI3K and CXCR-4, block tube formation, and suppress the levels of IL-8, VEGF, and angiogenesis in HMVEC cell lines [123].

### 2.6. Piperine

Piperine is a common alkaloid found in the Piperaceae family, particularly in the species *Piper nigrum*, which is known as black pepper and commonly used as a spice. Piperine possesses a variety of bioactivities, some of which include anticancer, antispasmodic, antidepressant, antioxidant, antiasthmatic, aggregating, and anxiolytic properties [124]. Piperine was reported to be the cause of dose-dependent killing of prostate cancer cells, particularly LNCaP and PC-3. The induction of apoptosis occurs by blocking the voltage-gated K+ current (IK) and arresting the cell cycle. This permanently stops the cell cycle. Ba and Malhotra published their results in 2018, and George et al. published their results in 2019 [125,126]. As Zeng and Yang reported in 2018, piperine was discovered to inhibit movement and cause programmed cell death in DU145 cells [127]. This was achieved by reducing the activities of phospho-Akt, phospho-mTOR, and MMP-9. According to Makhov et al. (2012), the use of piperine, which inhibits CYP3A4 activity, is associated with an increase in the anticancer efficacy of docetaxel in a mouse model of human CRPC [128]. This was observed when the two drugs were administered simultaneously. For DU145, PC-3, and LNCaP cells, piperine was discovered to induce apoptosis through the cleavage of PARP, activation of caspase-3, and the inhibition of PSA, a signal transducer and activator of transcription 3 (STAT-3) in the NF-κB signaling pathway [129]. Piperine reduced the activity of cytochrome p450 enzymes, including P-glycoprotein and CYP1B1, resulting in an increase in the effectiveness of docetaxel against taxane-resistant prostate cancer tumors. Cytochrome P450 enzymes play a key role in the hepatic metabolism of docetaxel, whereas P-glycoprotein is a multidrug-resistant protein widely known for its ability to limit the concentration of docetaxel in the body, as well as for its efficiency [130].

### 2.7. Lycopene

This bioactive compound is a carotenoid pigment present in significant amounts in various fruits and vegetables. Due to its antioxidant properties, lycopene helps reduce the risk of cardiovascular disease and skin damage. Recent studies by Gioti and Tenta (2015) and Pereira Soares et al. (2014) have shown that lycopene has chemopreventive properties and can induce cytotoxicity in prostate cancer cells via the mitochondrial apoptotic route [131,132]. Lycopene has been shown to inhibit the activity of Ras and HMG-CoA reductase, which in turn inhibits the growth of several cancer cells, including prostate cancer. Palozza et al. (2010) and Ivanov et al. (2007) found that apoptotic cell death was associated with the onset of cell cycle arrest and the deactivation of JNK, Akt, and NF-κB [133,134]. By inhibiting the activity of integrins, lycopene could inhibit the motility and infiltration of prostate cancer cells. In research conducted by Mirahmadi and colleagues (2020), it was discovered that lycopene induces apoptosis by increasing the expression of miR-let-7f1 and decreasing the expression of Akt [135]. Lycopene, as a chemopreventive compound, has been shown to reduce the malignancy of prostate cancer (PCa) by preventing the transformation of high-grade prostatic intraepithelial neoplasms into prostate cancer (PCa). This phenomenon has been proven by the results of clinical studies [136]. In 2005, the decline in malignancy was between 24 and 36 percent [136]. By reducing the IGF-1 level, Tjahjodjati et al. (2020) showed that lycopene was able to sensitize prostate cancer cells from Indonesian patients with a Gleason score of 6 [137]. Lycopene possessed anti-inflammatory effects by inhibiting the production of a variety of cytokines, including IL-6, IL- 8, IL-1 and TNF-, in PC-3, DU145, and LNCaP cell lines but also in a mouse model of tumor stress [138].

### 2.8. Quercetin

Quercetin is a flavonoid with five hydroxyl groups that exists in a range of fruits and vegetables, including onions, tomatoes, tea, and berries. The anticancer, antioxidant, immunomodulatory, and anti-inflammatory effects of this molecule are among the many therapeutic effects it possesses. Quercetin suppresses the proliferation of tumors in living organisms through reducing the expression of the lncRNA MALAT1 and regulating the PI3K/Akt signaling pathway [139]. This is achieved by preventing the growth and spread of the cells, as well as preventing the cells from transforming into a more invasive state. By regulating the PI3K/Akt signaling and androgen receptor pathway, resistance to docetaxel could be efficiently overcome in both in vitro experiments and in vivo experiments. Quercetin is responsible for inducing apoptosis and reducing the expression of PSA, TWIST-1, and P-glycoprotein in living organisms. In addition, it can overcome resistance to chemotherapy, which significantly increases the effectiveness of docetaxel [140]. Promoter methylation, PTEN, Bcl-2, IGF1, and cell cycle signaling were all affected by the combination of resveratrol and quercetin due to their combined effects. A decrease in IL-6, EGR3 and EGFR levels was also observed, and a concomitant increase in NKX3.1 and IGFBP7 levels was observed. The result was a reduction in tumor overload caused by apoptosis [141]. In both in vivo and ex vivo cancer models, the combination of quercetin and metformin resulted in caspase-dependent apoptosis, as well as reduced invasion and activity of Akt/PI3K, VEGF, and Bcl-2 [142]. Furthermore, the co-administration of these two drugs significantly reduced cancer cell growth. A study by Zhang et al. (2020b) found that it improved the effectiveness of paclitaxel in treating diseases, increased the arrest of cells in the G2/M phase, induced programmed cell death, caused endoplasmic reticulum stress, generated ROS, and was associated with an increased growth and movement of cells in both in vitro as and in vivo studies [143]. Quercetin was responsible for cell death as it passed through the mitochondrial/ROS pathway and triggered apoptosis. On the other hand, the activity of the tyrosine protein kinase-Met (C-Met) signaling pathway and PI3K/Akt signaling pathway decreased simultaneously. Experiments conducted in the laboratory showed that quercetin can reverse resistance to doxorubicin [144]. Research conducted by Tummala and colleagues in 2017 showed that combining enzalutamide, a next-generation antiandrogen, with another treatment enabled cells to eliminate drug-resistant prostate cancer cells efficiently [145]. To achieve this, the expression of certain genes was reduced. These genes included ARV7, AR, hnRNPA1, and AR-regulated genes, including UBE2C, FKBP5, NKX3.1, and PSA. The results of in vitro and in vivo experiments confirmed that this combination was beneficial. Moreover, research conducted by Zhao and colleagues in 2016 showed that quercetin nanomicelles could successfully induce apoptosis in colon cancer cells through in vitro and in vivo approaches [146].

Through its ability to inhibit the development of angiogenesis and the process of cancer cell metastasis to other areas of the body, quercetin reduced the amount of tumor tissue present. To achieve this goal, the synthesis of thrombospondin-1, a chemical that slows the formation of blood vessels, was increased [147]. Several cellular processes were inhibited by the drug, including the activities of the PI3k/Akt and EGFR signaling pathways and the expression of cyclin D1, N-cadherin, vimentin, and PCNA. This led to an improvement in excessive tumor growth caused by the chemical (MNU + T) in male Sprague Dawley rats and also reduced the growth of tumors [148]. Quercetin was discovered to inhibit cancer proliferation through the process of apoptosis, and the proliferative effects of the Akt, AR, and IGFIR signaling pathways were inhibited in order to achieve this [149].

### 2.9. Wedelolactone

*Eclipta alba* and *Wedelia chinensis* produce polyphenol wedelolactone upon extraction. Wedelolactone has a variety of pharmacological properties, and these properties include antiphospholipase, anticancer, antihypertensive, antidote, hepatotoxic, and anti-inflammatory effects against snake venom [150]. Wedelolactone exhibited promising anticancer effects on prostate cancer cells and tumor xenografts, as it inhibited c-Myc at both transcriptional and translational levels. It was also reported that it promoted cell death through apoptosis [65]. Furthermore, wedelolactone and enzalutamide were found to have functional synergy in inducing apoptosis in PCa cells. Based on the findings of Sarveswaran et al. (2012), wedelolactone induced programmed cell death in both androgen-dependent and -independent prostate cancer cells. This was achieved by activating caspase 3 and JNK while inhibiting PKC but not Akt. It has been found that the administration of a standardized herbal extract containing abundant wedelolactone, apelin, and luteolin inhibited the formation of tumors and the spread of metastases in a xenograft model consisting of DU145 and PC-3 [65]. In addition, it was shown that the combination of an herbal extract and docetaxel reduced the overall toxicity caused by docetaxel alone. This was achieved by suppressing the activation of NF-kB, as described by Tsai et al. in 2017 [151].

### 2.10. Plumbagin

Plumbagin is a naphthoquinone derived primarily from the roots of the *Plumbago rosea* plant and used in traditional Indian medicine [152]. It has been stated that plumbagin has a wide spectrum of therapeutic effects, including antibacterial, antiproliferative, antidiabetic, antimalarial, anticoagulant, and antifertility effects [152]. Plumbagin was found to reduce proliferation and invasion by inducing apoptosis in hormone-resistant prostate cancer DU145 cells, but did not have this effect on normal RWPE-1 epithelial cells. This was achieved by inhibiting the NF-kB and Akt signaling pathways [153]. In mice, the amount of xenograft produced by DU145 was also significantly reduced. Plumbagin was also found to induce apoptosis in DU145 and PC-3 cells in addition to xenograft regression with low toxicity [68]. Abedinpour et al. (2017), stated that the inclusion of plumbagin in the diet led to an increase in the effectiveness of ADT and, as a result, an increase in the survival rate of mice [154]. According to one study, a plumbagin nanoemulsion formulation showed a significant anti-proliferative effect against the PTEN-P2 PCa cell line in comparison with plumbagin alone [155]. It was found that plumbagin only stopped PTEN-P2-driven tumor proliferation in orchiectomized mice and not in normal mice, which means that dihydrotestosterone (DHT) is mostly made in the testes and stops prostate cells from dying [47].

### 2.11. Sulforaphane

The naturally occurring substance known as sulforaphane is an isothiocyanate obtained from the *Brassica oleracea* plant, which belongs to the Brassicaceae family. This compound occurs naturally in nature. Throughout history, many studies have highlighted the beneficial effects of cruciferous vegetables in treating different health problems, such as PCa. Researchers have found that certain vegetables contain a compound called sulforaphane (SFN), which has a strong impact on prostate cancer (PCa) [69]. It has also been shown that SFN can effectively limit the spread of cancer cells, their invasion of healthy tissue, and their ability to develop into stem cells. In addition, SFN has been shown to disrupt important signaling pathways in cancer cells, including the PI3k/Akt pathway. In addition, SFN influences the energy metabolism in cancers, particularly by interfering with glycolysis, the pentose phosphate pathway, and lipogenesis [46]. Using Hi-Myc and TRAMP mice, it has recently been reported that SFN treatment inhibited energy metabolism in PCa cells. This was observed through an increase in glycolysis, commonly known as the Warburg effect [156,157]. In 2018, Singh et al. showed that SFN had significant effects on the expression of key components of the glycolysis pathway, namely, lactate dehydrogenase A, hexokinase II, and pyruvate kinase M2 (LDHA, HKII, and PKM2, respectively), in LNCaP cells [156]. However, no such effect was observed in PC3 cells. SFN treatment resulted in the induction of lysosome-associated membrane protein-2 (LAMP-2) expression. In 22Rv1 and PC3 cells, LAMP2 RNA interference caused autophagic cell death, as Hahm reported in 2020 [158]. When using SFN-N-acetylcysteine (SFN-NAC) and SFN-cysteine (SFN-Cys) as treatment, PC3 and DU145 cells experienced apoptosis. This was achieved by interfering with microtubules and activating ERK1/2. It is worth noting that SFN-NAC had a longer half-life compared to SFN [159]. The inhibition of galectin-1 by SFN-Cys prevented the invasion of DU145 and PC3 cells. This was achieved by suppressing ERK1/2 phosphorylation, a process that facilitates invasion [160].

SFN administration resulted in significant repression of LNCaP and DU145 through the inhibition of hTERT activity, the catalytic component of telomerase-like proteins. Changes in post-translational histone alterations, which are commonly accompanied by an increased likelihood of prostate cancer recurrence, were responsible for this. More specifically, the regulatory components of the hTERT promoter region were involved in the dimethylation of histone H3 lysine four and acetylation of histone H3 lysine 18 [47]. Furthermore, SFN facilitates chromatin condensation by modifying the expression and recruitment of the chromatin compactor MeCP2, which represses hTERT. The expression of 100-long non-coding RNA (LINC0116) was reported to be suppressed in normal prostate epithelial cells (PRECs), LNCaP, and PC3 due to the presence of SFN [161]. According to Beaver et al. (2017), LINC0116 is known for its function in prostate cancer tumorigenesis, signaling metabolism, and cell cycle regulation, A double-blind, randomized, controlled clinical study involving 98 biopsies was reported. Compared to those who received a placebo, those given broccoli sprout extract (BSE), which is rich in SFN, exhibited a significant increase in the amounts of SFN and SFN isothiocyanates in plasma and urine [162]. In addition, these individuals had a differential expression of forty genes, such as ARLNC1 and AMACR, both of which have been linked to prostate cancer development [163].

### 2.12. Resveratrol (RES)

Resveratrol is a polyphenol that can be found in the stem of the *Vitis vinifera* plant after it has been dehydrated [164]. The RES trans isomer exhibits a wide spectrum of pharmacological potentials, including anticancer, anti-aging, cardioprotective, antioxidant, neuroprotective, anti-inflammatory, and immunomodulatory activities [164]. In 2013, Jasiski and colleagues published a study indicating that RES is responsible for apoptotic cell death in three cell types: PC3, DU145, and LNCaP [165]. In combination with actinomycin D, cytarabine methotrexate, taxol, and doxorubicin, the administration of RES resulted in an increase in the number of apoptotic cell deaths in prostate cancer cells. This was particularly the case when the active ingredients were administered together [165]. According to Gupta et al. (2011), this effect had been previously reported for a reduced expression of survivin and an increased intensity of apoptosis [166]. The study found that the sensitization of PC-3M-MM2 cells led to a reduction in the activities of IGF-1 and its receptor (IGF-1R), estrogen, ERK-1/2, and Akt. According to Athar et al. (2009), research on resveratrol has shown that it can inhibit the activity of NF-kB and cyclooxygenase2 (COX2) in prostate cancer cell lines. In addition, it has been observed to enhance signals that promote apoptosis [167]. The oral administration of resveratrol reduced the number of highly aggressive prostate cancer cells (PC-3M-MM2) in SCID mice. These cells lacked androgen receptors. To achieve this result, the inhibition of Akt and prostate cancer-related microRNA, such as miR21, and an increase in the levels of the PDCD4 tumor suppressor were implemented [168]. The antiapoptotic prosurvival pathway and the SphK1/S1P pathway, which is a product of the ERK1/2 pathway, were both blocked by RES, leading to the suppression of prostate cancer (PCa) both in vitro and in vivo [47]. The clinical testing of RES in individuals with benign prostatic hyperplasia showed a significant decrease in serum testosterone levels without causing prostate tumor growth [169]. A significant reduction in serum testosterone levels was observed. By reducing oxidative stress and enhancing apoptotic signals, the combination of RES and quercetin contributed to reduced carcinogenesis in the TRAMP model [141]. In a study conducted by Ye (2020), it was discovered that RES effectively inhibits the proliferation of prostate cancer cells and their programmed cell death. This was achieved by inhibiting the expression of the Akt signaling pathway, androgen receptor variant 7 (AR-V7), and the androgen receptor (AR) pathway [170]. In PCa cells, RES was found to block tumor invasion and migration by increasing the expression of TIMP2/3 and eleven translocation 1 (TET1) levels while simultaneously reducing the expression of MMP2/9 and TNF Receptor-associated factor 6 decreases (TRAF6)/NF-B/SLUG axis [47,171]. In 2020, RES reduced the development of prostatic fibrosis caused by inflammation. According to Hsieh and Wu’s 2020 research, RES inhibited the release of hepatocyte growth factor (HGF) by stromal cells, which in turn impedes invasion and the epithelial–mesenchymal transition (EMT) in prostate cancer (PCa) cells [172].

### 2.13. Triptolide

A substance called triptolide, which belongs to diterpenes, originates from the species *Tripterygium wilfordii*. Triptolide offers a broad spectrum of potent pharmacological actions, including the ability to inhibit carcinogenic, neurodegenerative, autoimmune, and inflammatory processes [173]. The chemical triptolide may trigger programmed cell death in both PC-3 and LNCaP cells. In addition, it has been reported to alleviate tumor development in a xenograft model formed by PC3 in mice [174]. According to the results of one study (Yuan et al., 2016), it could reduce the expression of MMPs, CD147, and caveolin-1, which in turn prevents invasion and migration in prostate cancer patients [175]. Triptolide inhibits the binding of RNA polymerase II, TFIIH, and AR to the target enhancer, resulting in an increased responsiveness of metastatic castration-resistant prostate cancer cells (mCRPCs) and xenograft models to enzalutamide treatment [174].

## 3. Marine-Derived Bioactive Compounds

**Figure 2:**
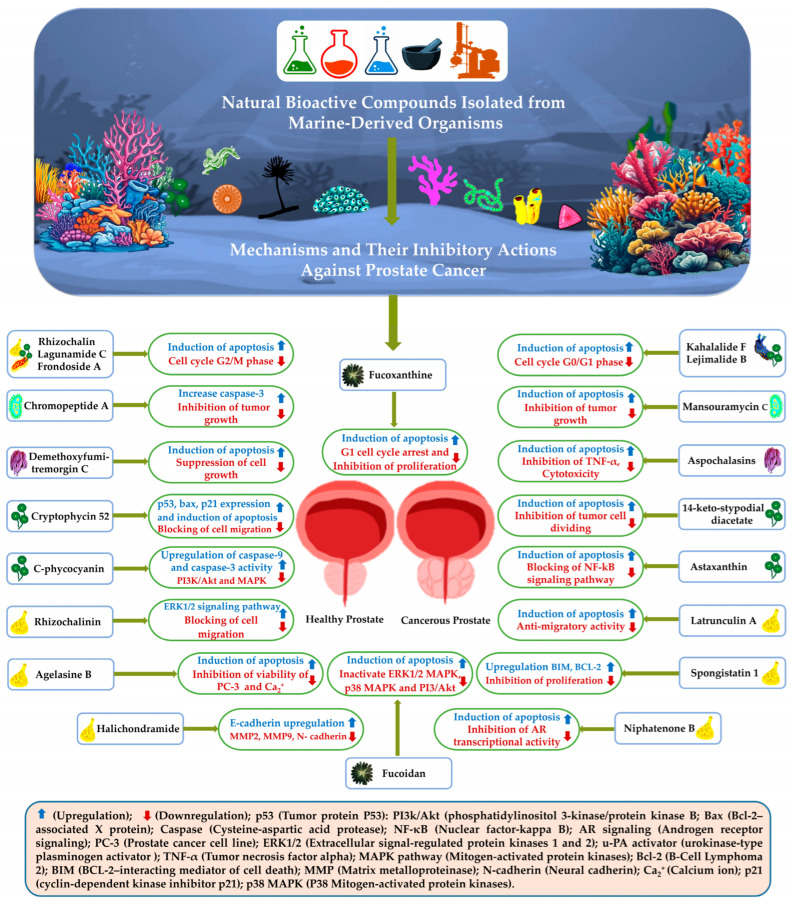
Marine bioactive compounds and their mechanism of action against prostate cancer.

It has previously been said that fifty percent of the drugs manufactured or discovered are formulated using chemical/biological structures derived from natural products (NPs) [176]. The vast majority of medicines are obtained from land-based sources. Due to the special chemical structure and biological activity they possess, the focus has currently shifted away from products produced on land and towards products based in the sea, namely marine natural products (MNPs) [177]. A total of 32 of the 34 phyla discovered on our planet are located in the ocean, which makes up most of the Earth’s surface and accounts for seventy percent of its size [178]. In marine ecosystems, there is a large variety of species per square meter, showing a wide range of diversity. Additionally, each area has a significant variation in biodiversity, with coral reefs having the highest diversity of all species combined. An overwhelming majority of researchers agree that the ocean holds a huge quantity of nature-based products that have never been explored before. Compared to natural products obtained from terrestrial sources, the bioactivity of substances taken from the marine environment is significantly higher. According to Rigogliuso et al. (2023) [179], 300 of these chemicals are registered as trademarks. Thirty-seven patents have been recorded in the United States and Europe, particularly relating to goods found in the deep sea. Most newly developed compounds are derived from soft-bodied invertebrates currently found in coral reefs [180]. New-generation drugs derived from sea sponges are being prepared for commercial development. There are lots of marine-based natural bioactive compounds available to manage prostate cancer, as shown in Table 2 and Figure 2.

### 3.1. Marine Bacteria

Microorganisms found in marine environments offer unique and valuable sources of efficient compounds that could be used in the fight against cancer. Due to the fact that marine microorganisms have the potential to be used in the development of medicines, researchers are fascinated by them. New anti-inflammatory compounds, such as manoalide, scytonemin, topsentins, and pseudopterosins, have been discovered in microorganisms found in marine environments [182]. Certain cancer cell lines, such as PC-3, are susceptible to the cytotoxic effects of these chemicals. Microbes from the mollusk *Elysia rufescens* are responsible for producing kahalalide F (KF), a chemical that has been reported to exhibit anticancer potential in both in vitro and in vivo. In particular, it has proven effective against many forms of solid tumors. During antiproliferative research in the laboratory, kahalalide F was found to exhibit activity against certain prostate cancer cells (DU-145 and PC-3). In contrast, it showed no activity against the hormone-sensitive LNCaP line [202]. KF has been shown to be effective against solid prostate tumors and has demonstrated its anticancer properties. During clinical trials, KF was administered intravenously to adult patients suffering from advanced or metastatic prostate cancer that was resistant to androgens. The administration of a dosage of 80 g/kg/day resulted in a partial response in a single patient. This was demonstrated by a reduction in PSA (prostate-specific antigen) levels of at least fifty percent over at least four weeks. There were five patients who showed evidence of stable disease. It is possible to safely administer KF by means of a one-hour infusion over a period of five days at a dosage of 560 g/kg/day, and the frequency of administration is once every three weeks [203].

On the other hand, solid-phase extraction and ethyl acetate were utilized in the research conducted by Hawas et al. in order to cultivate and extract the bacterium known as Streptomyces sp. Mei37. The bacterium was extracted from the muddy silt of Jade Bay in northern Germany. The researchers discovered five isoquinoline quinones, including the well-known 3-methyl-7-(methylamino)-5,8-isoquinolinedione, as well as four novel derivative compounds consisting of mansouramycin A-D [204]. All these nature-based compounds were tested on many cancer cells, including the PC3 and DU-145 prostate cancer cell lines. One of the most effective antibiotics was mansouramycin C, whose IC_50_ values were between 0.24 and 1.11 uM. A secondary metabolite was obtained from marine actinomycetes, particularly from the genus Streptomyces, and given the name Lu01-M. These actinomycetes were recovered from marine sediments in Taiwan at a depth of 400 m. Experiments were run using a series of prostate cancer cell lines, including PC3, DU145, and LNCaP cells, to investigate the effect of this chemical at different concentrations (from 0.78, to 12.5 g/mL) to be evaluated over a period of 24 to 72 h. The results showed that cytotoxicity was mediated by a number of different mechanisms, including the activation of necroptosis, apoptosis, and autophagy, arrest of the G2/M phase of the cell cycle, and DNA damage [205]. Chromopeptide A is a depsipeptide generated by the bacteria that is Chromobacterium sp., which is produced from marine sediments. This chemical has been demonstrated to have an intriguing potential for epigenetic remodeling by blocking the activity of histone deacetylases (HDACs 1, 2, 3 and 8). It is stated that all these HDACs are well known to be overexpressed in prostate cancer and are associated with a worse prognosis. The IC_50_ values for chromopeptide A are 2.08, 2.43, and 1.75 nmol/L, respectively, which indicates that it is capable of inhibiting the development of PC3, DU145, and LNCaP prostate cancer cells. Furthermore, it leads to cell cycle arrest in the G2/M phase in PCa cells by reducing the phosphorylation of both cdc2 and cdc25C, resulting in a PARP cleavage-associated increase in caspase-3 activity in three different cell lines examined. One study demonstrated that the administration of chromopeptide A (in the range between 1.6 and 3.2 mg/kg once per week for 18 days) through intravenous injection to mice that had received PC3 prostate cancer xenografts had the potential to inhibit the growth of tumor cells [182,183].

### 3.2. Marine Fungi

Marine fungi provide a variety of compounds which contain metabolites that possess significant biological activities, but research in this area is still quite limited. In one study, the effects of the marine fungus metabolite 1386A, which was taken from the South China Sea, were investigated with regard to the proliferation of androgen-independent cell lines, namely DU-145 cells. When DU-145 cells were incubated with 1386A for different time periods, such as 24, 48, and 72 h, IC_50_ values of 25.31, 8.62 and 4.79 mol/L, respectively, were achieved [192,206]. There is a possibility that this effect could be utilized to treat several neoplastic diseases, such as prostate cancer, either as a therapeutic intervention or as an additive in food. Demethoxyfumitremorgin C, a metabolite of the marine fungus *A. fumigatus*, was discovered to have a cytotoxic effect on PC-3 cells [184]. In a fascinating study of the bioactive compounds produced by marine gut fungi, researchers made a fascinating discovery. Aspochalasins were discovered in the digestive tract of the sea isopod known as Ligia oceanica. There is a group of cytochalasans that includes aspochalasins. These cytochalasans have specific characteristics, such as a macrocyclic ring, an isoindolone moiety, and a 2-methylpropyl side chain. It is stated that Aspochalasins have been found to possess various bioactivities, such as the inhibition of melanogenesis, inhibition of TNF-alpha, anti-HIV properties, and cytotoxicity [182]. Cytotoxicity evaluation was performed using the MTT method on PC-3 cells, a type of prostate cancer cells. The results of this group’s investigation revealed that apochalasin V had a modest activity that reached an IC_50_ value of 30.4 uM [192].

### 3.3. Marine Sponges

There is a significant amount of alkaloids found in marine sponges. For example, rhizochalin is a compound with biological activity that was first obtained from *Rhizochalina incrustata* (a sea sponge). One study found that rhizochalin has promising anticancer effects on human castration-resistant prostate cancer cells. The effect of rhizochalin was observed and the result showed that rhizochalin has the ability to inhibit autophagy, arrest the G2/M phase of the cell cycle, and induce apoptosis [188,207]. Moreover, the hydrolysis of rhizochalin produces a sphingolipid-like molecule called rhizochalinin (Rhiz). Rhiz demonstrated significant cytotoxicity on various human prostate cancer cells, including VCaP, LNCaP, DU145, 22Rv1, and PC-3. These effects were observed at relatively low micromolar doses. In many cases, aglycones tend to exhibit higher cytotoxicity compared to glycosides [187]. Studies have confirmed that Rhiz can inhibit cell migration in PC-3 cells. Additionally, the involvement of ERK1/2 was validated by the utilization of Western blot analysis. The statement that Rhiz-treated prostate cancer cells showed survival effects which may indicate a resistance mechanism was made [183]. In addition, the ethanol extracts of the sea sponge Haliclona spp. contain two important alkaloids called heliclonadiamine (HCA). These alkaloids exhibit strong cytotoxicity on the PC-3 cell line, resulting in a viability rate of 50% at a concentration of 100 µM [208]. The overexpression of regenerating liver phosphatase-3 (PRL-3) was effectively inhibited in these cells by HCA treatment. HCA promotes the activation of E-cadherin while decreasing the expression of N-cadherin, which is significantly overexpressed [209]. Latrunculin A, a macrolide molecule derived from *Negombata magnifica* (a red sea sponge), was found to inhibit the invasive properties of PC-3 cells [210]. Halichondramide, another macrolide compound from *Chondrosia corticata*, has been found to influence various biomarkers associated with prostate cancer. These biomarkers include MMP9, MMP2, N-cadherin, and E-cadherin, and the compound achieves this by modulating their gene expression and protein synthesis [189,192]. The presence of certain biomarkers can help determine prostate cancer metastasis. It was discovered that a macrocyclic lactone called spongistatin 1, which is generated from a marine sponge, can trigger programmed cell death and caspase-independent cell death, as assayed on DU-145 cell line [190]. Furthermore, spongistatin 1 affects both the microtubular complex and the anti-apoptotic protein MCL-1, which leads to an increase in the production of BIM, a member of the BCL-2 family that is responsible for promoting cell death. A genetic component known as BIM is responsible for the activation of apoptotic signaling pathways in prostate cancer. This component plays a vital role in the disease. These pathways are carried out by mitochondria and do not involve caspases [211]. The sea sponge-derived furanose ester terpene furospinosulin-1 was found to exhibit targeted antiproliferative properties against DU-145 cells in hypoxic environments [212]. The PC-3 cells were effectively suppressed by yardenone and sodwanone derived from Axinella sp. through the deactivation of HIF-1 [213]. This compound, known as Niphatenone B, is a glycerol ether that occurs naturally in crude extracts of *Niphates digitalis*. It was reported to play a role in the development of castration-recurrent prostate cancer. It promotes the growth of LNCaP cells but does not have the same effect on PC-3 cells. For this reason, there is currently a lack of effective AR protein support to combat targeted anti-proliferation efforts. The activation function 1 region of the N-terminal domain (NTD) of the AR protein was found to exhibit a substantial affinity for niphatenone B, as observed in [214]. A new compound, agelasine B, was discovered in the sea sponge that is *Agelas clathrodes*. Scientific evidence clearly indicates that this chemical has a high impact on the survival of PC-3 cells. This leads to a relevant reduction in the concentration of Ca^2+^ in PC-3 cell lines and triggers DNA fragmentation [192].

### 3.4. Marine Algae

Marine algae, including blue, green, and brown forms, include a range of bioactive chemicals utilized in the manufacture of pharmaceuticals. Numerous bioactive chemicals have been identified in marine algae and are utilized in several types of cancer, including prostate, breast, and colon cancer, among others. The details of all these algae are below.

#### 3.4.1. Cyanobacteria

The prokaryotic organisms which are known as cyanobacteria or sea blue algae can be discovered in a variety of habitats. There is a macrocyclic molecule known as cryptophycin 52 that can be found in nature and contains anticancer properties. It is generated by marine cyanobacteria belonging to the *Nostoc* spp. [215]. Lagunamide C, a new cyclic depsipeptide, was also reported and isolated from a cyanobacterium species, *L. majuscula*. This cyanobacterium was acquired from Pulau Hantu Besar, which is located in Singapore. The effectiveness of Lagunamide C against PC3 cells was determined to be 2.6 nM, as demonstrated by its IC_50_ value. In addition to this, it possesses significant antimalarial properties [193]. Moreover, Lagunamide C is responsible for causing cell cycle arrest, specifically in the G2/M phase. Cytotoxic peptides, such as dolastatins, which are generated by *Dolabella auricularia*, are also responsible for this, as are their synthesized equivalents, such as dolastatin, in addition to their natural counterparts [216]. Marine cyanobacteria-derived compounds have the ability to disrupt caspases and set off the chain of events that ultimately results in the death of cells. Among the caspases that are involved in the process of programmed cell death in prostate cancer cells, caspase-3 is the most prevalent. According to earlier research, the anticancer properties of C-phycocyanin (C-PC), which is derived from the *Limnothrix* sp. cyanobacterium, were proven. When compared to the use of topotecan (TPT) alone at its full dose, our data reveal that tumor cells were destroyed at a substantially higher rate when a combination of C-PC and a mere 10% of the regular dosage of the anticancer medicine TPT was utilized. Furthermore, when these two compounds were applied simultaneously, we found that the amount of caspase-9 and caspase-3 activities was significantly increased [195]. When it comes to prostate cancer, the BCL-2 protein family is an essential component in the process of controlling apoptosis. In several lines of prostate cancer, including PC-3, LNCaP, and DU-145, it has been demonstrated that cryptophycin 52 is capable of activating the phosphorylation of BCL-xL and BCL-2 [194]. A marine macrolide known as Iejimalide B was initially obtained from the tunicate Eudistoma cf. rigida, which is found in the ocean. For doses in the nanomolar range, iejimalide B had activity on both PC-3 and LNCaP cell lines; nevertheless, the effects of the compound on these two cell lines were very different from one another. Furthermore, iejimalide B administration at dosages lower than 30 nM resulted in the termination of the cell cycle during the G0/G1 phase. Furthermore, the administration of doses that were equal to or higher than 50 nM led to the death of LNCaP cells. Neither of these doses induced apoptosis in PC-3 cell lines after more than 72 h [217].

#### 3.4.2. Chlorophytes

The term Chlorophyta refers to a type of algae that are green in color and are formed through photosynthesis. The vast majority of algae are classified as chlorophytes. They are found in marine environments and categorized as sources of important nutrients, including minerals and vitamins. The ability of these substances to combat prostate cancer is another area in which they have shown promise. Extensive research has been conducted on green algae, which has resulted in the identification of a significant number of chemicals that have the potential to suppress the proliferation of cancer cells. As an illustration, the chemical known as 14-keto-stypodiol diacetate (SDA), which is derived from the sea alga known as *Stypopodium flabelliforme*, might suppress both the proliferation of cell lines and their neoplastic activity in DU-145 cells. According to the results of research, this newly discovered molecule, which is produced from a natural component of the marine environment, is responsible for preventing tumor cells from dividing. This result may be associated with changes in typical microtubule production [192,218]. Furthermore, the influence of this molecule on protease secretion and its ability to invade in vitro are properties associated with cells derived from metastatic areas. This molecule is important for both properties [218,219]. The incorporation of micromolar concentrations of SDA significantly reduced the release of plasminogen activator (u-PA) and alleviated DU-145 cells across a Matrigel-coated membrane [192]. The carotenoid astaxanthin, which potently activates antioxidants and apoptosis by blocking NF-kB, receives a significant portion of its supply from *Haematococcus pluvialis*, an abundant source of the carotenoid. Astaxanthin has an antiproliferative effect on various prostate cancer cell lines [220]. Kahalalid (KF), a remarkable bioactive chemical, is produced from the plant *Elysia rufescens*. According to current research, the Bryopsis species is the main source of KF. When applied to a cell line derived from prostate cancer, this chemical triggers oncogenesis. Therefore, KF is responsible for permeability in lysosomes and cell membranes, as well as for inducing apoptosis by inhibiting the PI3K/AKT signaling pathways [192,193].

#### 3.4.3. Phaeophyta

Phaeophyta, also called brown algae, are capable of producing complex diterpenoids and metabolites formed by combining aromatic chemicals with terpenoid molecules. Previous research has demonstrated that a significant proportion of these compounds have potent antibiotic, antiviral, antifungal, and anticancer effects [221]. The brown algae species *Undaria pinnatifida* and *Cladosiphon novaecaledoniae*, as well as other species, are suitable sources for their extraction. PC-3 cells were inhibited by fucoidan due to its ability to stimulate both intrinsic and extrinsic apoptosis [222]. The subsequent deactivation of the extracellular p38 mitogen-activated protein kinase (p38 MAPK) and signal-regulated kinase MAPK (ERK1/2 MAPK) occurred simultaneously with apoptosis. In addition, phosphatidylinositol 3-kinase (PI3K)/Akt was inactivated. Furthermore, the administration of fucoidan led to an increase in the expression of the p21Cip1/Waf gene. As a result, fucoidan reduced the expression of E2F-1 proteins, which are involved in cell cycle regulation while increasing the activity of the Wnt/-catenin signaling pathway [198,222]. In PC-3 cells, a decrease in the amount of -catenin, as well as a decrease in c-MYC and cyclin D1 expression, were observed as a result of the activation of the GSK-3 protein. When it comes to the EMT process of cancer cells, TGFRs and TGF play a key role. As shown in a study, fucoidan was able to effectively correct the morphological changes caused by the TGFR-mediated epithelial-to-mesenchymal cell transition. For this reason, fucoidan has been shown to increase the expression of epithelial markers in prostate cancer cells while decreasing the expression of mesenchymal markers. In addition, it reduces the expression of the transcriptional repressors Twist and Snail/Slug [223].

### 3.5. Marine Diatoms

Only a small number of naturally occurring bioactive chemicals have been extracted from diatoms, even though diatoms are abundant everywhere. An essential marine compound called fucoxanthin was isolated from Sargassum sp., and it is currently used in prostate cancer treatment. Fucoxanthin has been shown to inhibit the development of LNCaP cells [199]. Evidence of a suppressive effect on growth was demonstrated by the stimulation of G1 and GADD45A cell cycle arrest. Fucoxanthin, a naturally occurring molecule, was found to be highly conjugated, and according to research, it is generally not associated with toxicological outcomes when it is used as an anticancer compound in the treatment of prostate cancer [224]. It was reported that when white Leghorn chickens were given the brown algae *F. serratus*, the fucoxanthin they ingested was deacetylated in their intestines and subsequently transported through their bloodstream. For this reason, fucoxanthinol, considered one of the most important carotenoids, was discovered in their egg yolk [225]. The biotransformation of fucoxanthinol in ICR mice was the subject of a study by Asai and his collaborators. They found a metabolite discovered in the tunicate *Amaroucium pliciferum* that had not previously been recognized [226]. Amarouciaxanthin A was the name of this particular metabolite. PC-3 cells experienced a reduction in viability when exposed to amarouciaxanthin A and fucoxanthinol. The dosages of fucoxanthin, fucoxanthinol, and amarouciaxanthin A that were found to be 50% inhibitory were 3.0, 2.0 and 4.6 M, respectively, as reported in references [192,226]. However, there are only a few studies on this topic.

### 3.6. Holothurians

Over the course of several millennia, the aquatic invertebrates known as holothurians, also known as sea cucumbers, have been used in traditional Asian medication. The triterpene glycoside known as frondoside A (FrA) was originally derived from edible sea cucumber (*Cucumaria frondosa*) extract. In human prostate cancer cells, the chemical known as FrA has shown significant levels of efficiency while causing little damage. This has even been confirmed in cell lines resistant to conventional treatments [200]. The molecule has unique properties that trigger programmed cell death while simultaneously terminating the cell cycle and blocking autophagy, which is essential for survival. In addition, there is a possibility that it may inhibit immune responses. Prostate cancer cells were inhibited by using 12-MTA. The use of PI labeling showed that 12-MTA was responsible for the death of PC-3 cells by inducing apoptosis, a process in which caspase-3 may have been involved. In amounts considered biologically important, 12-MTA might specifically reduce the synthesis of 5-hydroxyeicosatetraenoic acid, a byproduct of 5-lipoxygenase. There is a possibility that this drug could potentially serve as a novel and complementary treatment for certain cancers, such as prostate cancer [192,227].

## 4. Integration of Plant- and Marine-Derived Bioactive Compounds with Conventional Therapies Against Prostate Cancer

In recent times, the integration of bioactive compounds derived from plant and marine sources with conventional therapies has emerged as a promising strategy in prostate cancer management. These natural compounds bear antioxidative, anti-inflammatory, and antiproliferative properties that could alleviate the therapeutic efficacy while reducing the adverse effects of standard treatments.

The potential of plant-derived bioactive compounds, such as flavonoids, alkaloids, polyphenols, etc., in prostate cancer treatment has been reported in several studies [10,228]. For instance, epigallocatechin-3-gallate (EGCG), a major catechin found in green tea, has been reported to suppress prostate cancer progression by inhibiting androgen receptor signaling, which is a crucial pathway targeted in androgen deprivation therapy (ADT) commonly used in prostate cancer treatment [87,229]. Similarly, curcumin, a polyphenol from turmeric, in combination with docetaxel, a standard chemotherapeutic agent, inhibits the proliferation of and induces apoptosis in prostate cancer cells [230,231,232]. Combined treatment with curcumin and docetaxel has been reported to modulate the expression of RTKs, PI3K, phospho-AKT, NF-kappa B, p53, and COX-2, which signifies the potential of curcumin to serve as a potential therapeutic contender in enhancing the efficacy of docetaxel in prostate cancer treatment [230]. Likewise, soy-derived isoflavones such as genistein and daidzein have demonstrated anticancer activity by modulating estrogen and androgen receptor pathways critical in prostate cancer development [233]. Genistein could induce apoptosis and inhibit the activation of the antiapoptotic protection factor, NF-κβ, in prostate cancer cells [234]. Moreover, it has been reported to inhibit the growth in prostate and other cancer cells by the upregulation of p21cip1/waf1 and a concomitant decrease in cyclin B, resulting in G2/M arrest [235]. Though several studies have acknowledged the role of soy isoflavones in prostate cancer treatment, there are still many gaps that are required to be investigated in depth to properly understand how soy isoflavones regulate cell biology relevant to prostate cancer management [236].

Marine-derived bioactive compounds also present significant therapeutic potential. Among marine sources, bioactive compounds extracted from seaweed have gained significant attention in the past two decades for developing novel anticancer compounds with greater efficacy and specificity while having fewer side effects [237]. Examples include polysaccharides such as fucoidans and laminarins extracted from brown seaweed, which have displayed anti-tumor and immune-modulatory effects against prostate cancer [198,238,239]. Fucoidan has been reported to enhance the efficacy of chemotherapeutic agents by inducing apoptosis and inhibiting angiogenesis in prostate cancer cells [198]. It was observed to modulate protein expression associated with apoptosis through the PI3K/Akt and MAPK signaling pathways [198]. In combination with Nivolumab, fucoidan compounds increased the effects of Nivolumab on prostate cancer cells by enhancing the activity of human immune cells and showed direct cytostatic effects on PC3 cells, reducing cancer cell numbers; PBMCs exhibited cell-killing activity [240]. Similarly, low-molecular-weight fucoidan, when combined with an oligonucleotide aptamer (GroA, AS1411), a cell surface Nucleolin inhibitor, significantly improved the anti-proliferative effect of GroA, as it decreased cancer cell growth and viability and increased cell death [241]. Marine peptides like kahalalide F, derived from marine mollusks, have shown selective cytotoxic effects on prostate cancer cells [181,182]. This dipsipeptide disturbs lysosomal function and induces cell death by intracellular acidification. Currently, clinical trials exploring their therapeutic applications are underway [242].

The combined use of these bioactive compounds with conventional therapies has several advantages. They can potentiate the cytotoxic effects of chemotherapy and radiotherapy while reducing treatment-induced oxidative stress and inflammation. Moreover, these natural compounds often target multiple signaling pathways involved in cancer progression, thus addressing tumor heterogeneity—a major challenge in prostate cancer treatment. Despite their promise, challenges such as poor bioavailability, pharmacokinetics, and variability in patient responses must be addressed through advanced formulation strategies, including nanotechnology-based delivery systems. Future research and clinical trials are essential to validate these findings and establish optimized treatment protocols for incorporating plant- and marine-derived bioactive compounds into prostate cancer therapy.

## 5. Nanoparticle-Based Delivery of Bioactive Compounds Derived from Plant Extracts and Marine Resources for Prostate Cancer Therapy

In recent times, the synthesis of nanoparticles using bioactive-rich plant extracts and marine resources has emerged as a promising approach to enhance the delivery and therapeutic efficacy of bioactive compounds in prostate cancer treatment [243,244]. Various setbacks associated with conventional treatments and natural bioactives, such as poor bioavailability, rapid metabolism, non-specific distribution, inadequate aqueous solubility, chemical instability, unsatisfactory circulation time, etc., could be effectively overcome by nanoparticle-based delivery systems [244,245]. The encapsulation of bioactive compounds in nanocarriers provides multiple benefits, such as targeted delivery to cancer cells, improved drug stability, and controlled release, thereby enhancing therapeutic outcomes while minimizing the side effects [243,246].

Plant-extracted bioactive compounds could be loaded onto the nanoparticles to facilitate their site-specific delivery, and they could be used as a reducing and stabilizing agent while synthesizing nanoparticles [247,248,249]. Several studies have successfully demonstrated the green synthesis of varied nanoparticles from plant extracts rich in bioactive compounds, such as polyphenols, flavonoids, and alkaloids, and their therapeutic applications [250,251,252]. For instance, green synthesis methods using extracts from turmeric, green tea, and other medicinal plants have been used to create gold and silver nanoparticles that exhibit enhanced anticancer activity [253,254,255]. Several studies have reported the synthesis of nanoparticles loaded with curcumin from plant extracts which have demonstrated significant cytotoxic effects against prostate cancer cells by improving curcumin’s solubility, bioavailability, and intracellular uptake. Yallapu et al. (2014) investigated the efficacy of poly(lactic-co-glycolic acid) (PLGA) nanoparticles loaded with curcumin (PLGA-CUR NPs) in inhibiting prostate cancer cell growth both in vitro and in vivo [256]. The results demonstrated that PLGA-CUR NPs effectively inhibited prostate cancer cell proliferation through lysosomal activity, apoptosis, inhibition of androgen receptor (AR), and nuclear β-catenin activity. Additionally, PLGA-CUR NPs modulated the expression of microRNAs miR-21 and miR-205 and showed significant prostate tumor-specific targeting in a xenograft mouse model using PSMA-PLGA-CUR NPs. Recently, Keshavarz Shahbaz et al. (2024) summarized various experimental animal studies demonstrating curcumin’s potent preventative actions against a wide variety of tumor cells at various stages, emphasizing the role of nanoparticle formulations in enhancing curcumin’s bioavailability and therapeutic effectiveness [257]. Similarly, green tea polyphenol-based nanoparticles have been reported to efficiently deliver catechins to prostate cancer cells, enhancing their therapeutic effects by protecting them from rapid degradation in the body. Khan et al. (2014) synthesized chitosan nanoparticles encapsulating epigallocatechin-3-gallate (Chit-nanoEGCG) and investigated their efficacy for the treatment of prostate cancer [258]. The findings revealed a significant inhibition of tumor growth and increased prostate-specific antigen levels after treatment with Chit-nanoEGCG. In tumor tissues of mice treated with Chit-nanoEGCG, significant induction of poly (ADP-ribose) polymerases cleavage was reported. In addition, an increase in the protein expression of Bax with a concomitant decrease in Bcl-2, an activation of caspases, and a reduction in Ki-67 and proliferating cell nuclear antigen were observed. Siddiqui et al. (2016) prepared chitosan nanobioconjugates encapsulating EGCG and surface-functionalized them with A10 2′-fluoropyrimidine RNA aptamers, employing a targeted uptake approach for improving the efficacy of EGCG for PCa management [259].

Marine resources also offer a novel platform for nanoparticle synthesis, utilizing compounds derived from seaweed, marine sponges, and marine peptides [260,261,262]. For instance, fucoidan, a sulfated polysaccharide extracted from brown seaweed, has been used to produce nanoparticles capable of delivering bioactive molecules to prostate cancer cells. Boo et al. (2013) studied the anticancer effect of fucoidan obtained from Undaria pinnatifida in PC-3 cells and revealed that fucoidan treatment induces intrinsic and extrinsic apoptosis pathways via the activation of ERK1/2 MAPK, the inactivation of p38 MAPK and the PI3K/Akt signaling pathway, and the downregulation of the Wnt/β-catenin signaling pathway in PC-3 prostate cancer cells [222]. Another study investigated the combination of anti-angiogenic drugs with seaweed-derived fucoidan [263]. The findings suggested that fucoidan inhibited angiogenesis and induced cell cycle arrest in cancer cells. The combination therapy showed an enhanced anticancer potential, indicating that fucoidan can synergize with other therapeutic agents to improve treatment outcomes [263]. Similarly, marine peptides such as kahalalide F (KF) have demonstrated significant anticancer properties, including selective cytotoxicity towards prostate cancer cells (Suárez et al. 2003). To enhance the therapeutic efficacy and selectivity of KF, researchers have explored its conjugation with nanocarriers. A study published in Bioconjugate Chemistry investigated the conjugation of KF with gold nanoparticles (GNPs) [264]. The results indicated that KF-GNP conjugates exhibited enhanced in vitro antitumoral activity compared to free KF, suggesting that the nanoparticle-based delivery system improved the targeting and effectiveness of KF against cancer cells [264].

By combining the therapeutic potential of bioactive compounds with nanotechnology, these approaches not only amplify anticancer efficacy but also create an intersection between traditional herbal medicine and modern technological advancements [244,265]. Nanoparticles derived from natural resources offer a biocompatible and sustainable alternative for prostate cancer therapy, with ongoing research focusing on optimizing nanoparticle formulations, improving targeting specificity, and conducting clinical trials to translate these findings into clinical practice [244]. This innovative integration of nanotechnology with bioactive compounds opens new avenues for more efficient and safer prostate cancer treatments.

Additionally, we examined government-based online data (clinicalTrials.gov) to identify certain plant- and marine-based bioactive compounds that have undergone clinical investigation related to prostate cancer; the results are shown in Table 3. We found that only a limited number of plant-based bioactive compounds have progressed to clinical investigation, but no marine-based bioactive compounds have reached this stage, and they are still recruiting volunteers to initiate the clinical trials.

## 6. Regulatory Norms Related to Plant- and Marine-Derived Bioactive Compounds in the Management of Prostate Cancer

The regulatory standards for plant- and marine-based bioactive compounds utilized in the management or treatment of prostate cancer differ from country to country and are typically supervised by the governmental authorities that regulate pharmaceuticals, supplements, and natural goods. Below is a summary of the typical components of such rules:

(a) Food and Drug Administration (FDA): The FDA oversees dietary supplements containing plant- and marine-derived bioactive substances in accordance with the Dietary Supplement Health and Education Act (DSHEA) of 1994. According to this legislation, manufacturers bear the responsibility for guaranteeing the safety of their products; nevertheless, the FDA does not authorize dietary supplements prior to their sale. Moreover, clinical investigations, traditional knowledge, and expert opinion help supplement manufacturers estimate the doses of plant-based ingredients. The FDA requires accurate supplement labeling, including ingredient listings and serving sizes, but does not regulate dosages. However, before being licensed by the FDA as a novel medication, a plant-based bioactive molecule must undergo clinical trials to confirm its safety, efficacy, and dosage. The FDA approves drug dosages based on clinical trial data on pharmacokinetics, toxicity, efficacy, and other considerations.
(b) European Medicines Agency (EMA): The EMA would also regulate these substances according to how they are meant to be used. Drugs that are meant to treat prostate cancer must go through clinical studies, while nutritional supplements are subject to various regulations.
(c) Therapeutic Goods Administration (TGA): The TGA regulates plant and marine-based bioactive chemicals, including complementary medicines and prostate cancer treatments, in Australia. Plant-based substances must have scientifically credible clinical evidence of their safety and efficacy to treat prostate cancer, especially if making specific therapeutic claims. Less stringent evidence may be needed if the substance is used for prostate health, but the TGA will still control claims and safety.
(d) Indian Ministry of AYUSH: The AYUSH Ministry of India promotes scientific validation, safety guidelines, and clinical research for plant- and marine-based bioactive substances to treat prostate cancer. Under the Drugs and Cosmetics Act, herbal products must meet strict quality control criteria for efficacy and safety. Clinical evidence is needed to approve and include plant-based medicines in prostate cancer therapy methods. Ayurvedic, Unani, Siddha, and homeopathic systems also study natural bioactive substances for integrated cancer care.

Significant scientific evidence is required for the utilization of plant- and marine-based bioactive compounds as prostate cancer therapies. This generally encompasses the following:

Preclinical studies: laboratory and animal investigations that assess a compound’s efficacy in suppressing prostate cancer cell proliferation or augmenting the immune response.

Clinical trials: studies using human subjects to determine safety, dosage, and effectiveness. Clinical study phases encompass safety assessment (Phase I), efficacy evaluation (Phase II), and extensive validation (Phase III).

Regulatory agencies mandate that these trials adhere to Good Clinical Practice (GCP) standards to guarantee patient safety and the integrity of the results.

Overall, the current review provides an overview of various nature-derived compounds and their potential beneficial effects against prostate cancer. It discusses several bioactive molecules derived mainly from plants, marine organisms, and other natural sources, as well as their mechanisms of action in inhibiting prostate cancer progression, inducing apoptosis, and counteracting drug resistance. Furthermore, our study discusses the challenges in early detection and the limitations of current screening methods, such as prostate-specific antigen (PSA) testing.

Our review deciphers and focuses on the anticancer properties of various natural compounds, including apigenin, epigallocatechin-3-gallate (EGCG), genistein, curcumin, ellagic acid, piperine, lycopene, quercetin, wedelolactone, sulforaphane, resveratrol, and triptolide, which are categorized as plant-based natural products. Additionally, some marine-derived compounds, such as those from sponges, cyanobacteria, fungi, and algae, are studied to investigate their promising anticancer effects against prostate cancer cells. Nevertheless, some of the most effective compounds include polyphenols such as EGCG, quercetin, and resveratrol, as they are potent factors that display anti-proliferative, anti-inflammatory, and (pre)-apoptotic effects and inhibit prostate cancer growth. Meanwhile, marine-based compounds such as astaxanthin, fucoidan, fucoxanthin, and spongistatin demonstrate considerable potential in preclinical studies for suppressing prostate cancer cell proliferation, causing apoptosis, and possibly augmenting the efficacy of further therapies. Nonetheless, additional study, especially clinical trials, is required to comprehensively determine their efficacy and safety for human application in prostate cancer therapy. While these compounds are promising and have potent beneficial effects, their efficacy depends on the specific prostate cancer type and stage, as well as some personalized conditions which require medical supervision.

## 7. Conclusions

The exploration of plant- and marine-derived bioactive compounds holds immense potential for advancing prostate cancer treatment strategies. This manuscript highlights a diverse range of compounds, such as apigenin, epigallocatechin-3-gallate, curcumin, resveratrol, and lycopene from plant sources, and kahalalide F, fucoxanthin, spongistatin 1, and astaxanthin from marine sources. These compounds are known to exhibit promising anticancer properties through their ability to modulate critical molecular pathways, including apoptosis, angiogenesis, and metastasis. These bioactives represent a valuable reservoir for drug discovery and for developing functional foods and nutraceuticals tailored for prostate cancer management, prevention, and therapy. The development of novel functional foods and nutraceuticals derived from these compounds can offer a complementary approach to conventional treatments, emphasizing the prevention and holistic management of cancer. By incorporating these bioactives into dietary interventions, it may be possible to reduce cancer risk, improve patient outcomes, and enhance patient quality of life with minimal side effects. Functional foods enriched with bioactive compounds such as lycopene, curcumin, and fucoxanthin could serve as accessible and sustainable dietary solutions, particularly in populations at high risk for prostate cancer. Nutraceutical formulations, meanwhile, can provide standardized doses of these compounds in a convenient form, enabling better compliance and targeted delivery of therapeutic benefits.

Future research should prioritize comprehensive in vivo and clinical studies to elucidate the pharmacokinetics, bioavailability, and synergistic effects of these compounds. Advances in encapsulation technologies, such as nanoparticles, liposomes, and biopolymer-based delivery systems, could further enhance the stability, absorption, and bioactivity of these agents in functional foods and nutraceutical products. Moreover, integrating these findings with precision nutrition and personalized medicine approaches could pave the way for tailored dietary and therapeutic interventions that align with individual genetic and metabolic profiles. This work underscores the importance of continuing to explore natural bioactives as a sustainable and versatile avenue for combating prostate cancer. By bridging the gap between basic research and translational applications, these efforts can contribute significantly to the global fight against cancer, offering innovative solutions for prevention, management, and treatment.

## Figures and Tables

**Table 1:** Collection of bioactive phytocompounds and their therapeutic applications in the prevention and management of prostate cancer.

Bioactive Compound	PubChem CID	Plant Species Name/Family name	Biological Functions	Signaling Pathway Downregulation Against Prostate Cancer	Signaling Pathway Upregulation Against Prostate Cancer	Mechanisms That Inhibit Prostate Cancer Cell Growth	References
Apigenin	6322	Anthemis species and Asteraceae family	Anticancer, neuroprotective, antioxidant, antiviral, anti-inflammatory, and antimetastatic activities	PI3K/Akt pathway and Hedgehog (Hh) signaling pathway Proteasomal function Glucose absorption, invasion, or movement Angiogenesis in prostate cancer cells promotes their stemness	PCa stem cells that have undergone extrinsic apoptotic cell death	Induction of apoptosis, cell cycle arrest, inhibition of angiogenesis	[46,47]
Epigallocatechin-3-gallate	65064	*Camellia sinensis* (L.) Kuntze. Theaceae family	Anticancer, antioxidant, antidiabetic, cardioprotective, and neuroprotective effects	Androgen receptor signaling, PI3K/Akt pathway, Hedgehog axis, lipogenesis, invasion, and motility	Apoptotic cell death	Induction of apoptosis, cell cycle progression	[48,49]
Genistein	5280961	Leguminosae family	Anticancer, antioxidant, anti-inflammatory, antiangiogenic, and anti-proliferative activities	EGFR levels and activity in AR signaling, NF-kB, PI3K/Akt, Hh, and Wnt axes, carcinogenic miRNAs Proteosomal involvement Lipogenesis Mobility/Invasion Metastasis of the bones PCa cell stemness	microRNAs Apoptotic cell death Oncosuppressive	Inhibition of the production of androgens, Induction of apoptosis	[50,51]
Curcumin	969516	*Curcuma longa* L. Zingiberaceae family	Antioxidant, anticancer, neuroprotective, and anti-inflammatory properties	PI3K/Akt, Hh, Wnt axis Bone metastasis AR signaling Testosterone levels PCa cell stemness Glutaminolysis Invasion/motility Oncogenic miRNAs	ROS production miRNAs Programmed-necrotic cell death ER stress Oncosuppressive Apoptotic cell death	Induction of apoptosis, cell cycle arrest, autophagy, inhibition of the formation of cancer-associated fibroblasts, inhibition of androgen receptor	[52,53]
Ellagic acid	5281855	Vaccinium species and Rubus species, family Ericaceae and Rosaceae	Anticancer and antioxidant properties	p-Akt/PI3K and CXCR-4, IL-8, VEGF, and angiogenesis in HMVEC cells	ROS production Apoptotic cell death	Induction of apoptosis, cell cycle arrest, inhibition of angiogenesis	[54,55]
Piperine	638024	*Piper nigrum* L. Piperaceae family	Anticancer, antispasmodic, antidepressant, antioxidant, antiasthmatic, aggregating, and anxiolytic properties	phospho-Akt/phospho-mTOR/MMP-9, STAT-3, PSA NF-κB	Apoptotic cell death	Induction of apoptosis, cell cycle arrest	[47,56]
Lycopene	446925	Tetraterpene carotenoid family; found in red fruit and vegetables	Antioxidant, anticancer, cardioprotective, anti-inflammatory	AR signaling, NF-κB pathway IGF-1 level JNK pathway Akt pathway	Apoptotic cell death miR let-7f1 expression	Induction of apoptosis, block of integrins	[57,58,59]
Quercetin	5280343	Labiatae or Compositae families	Anticancer, antioxidant, anti-inflammatory, antiviral, antihypertensive, and immunomodulatory properties	EGFR levels and their activity AR-V7 activity Endothelial cell growth Microvessel sprouting PI3K/Akt, Hh, Wnt axis PCa cell stemness AR signaling Invasion/motility Lipogenesis	Apoptotic cell death ER stress	Induction of apoptosis, cell cycle arrest, inhibition of angiogenesis	[60,61,62]
Wedelolactone	5281813	*Eclipta alba* (L.) *Hassk* and *Wedelia chinensis* (L.) Juss. Family Asteraceae	Anti-phospholipase, anticancer, antihypertensive, antidote, hepatotoxic, and anti-inflammatory properties	c-Myc NF-kB signaling	Apoptotic cell death JNK and caspase 3 level	Induction of apoptosis	[63,64,65]
Plumbagin	10205	*Plumbago rosea* L. Plumbaginaceae family	Anticancer, antimicrobial, antifertility, antidiabetic antimalarial, and anticoagulant	Akt and NF-κB signaling pathway	Generation of ROS Apoptotic cell death ER stress	Induction of apoptosis	[66,67,68]
Sulforaphane	5350	*Brassica oleracea* L. Brassicaceae family	Antitumor, antimicrobial, antioxidant, and anti-inflammatory activities	PI3K/Akt, NF-kB axis Lipogenesis and lipid-dependent metabolism Glycolysis Penthose Phosphate shunt Metastasis promoters AR function PCa cell stemness	Generation of ROS Apoptotic cell death	Induction of lysosome-associated membrane protein-2 (LAMP-2) expression, induction of apoptosis, inhibition of galectin-1, inhibition of human telomerase reverse transcriptase (hTRET)	[69,70]
Resveratrol	445154	*Vitis vinifera* L. Vitaceae family	Anticancer, anti-aging, cardioprotective, antioxidant, neuroprotective, immunomodulatory, and anti-inflammatory activities	NF-kB and Hh signaling AR signaling Glutaminolysis Oncogenic miRNAs EGFR levels and activity Glucose fermentation	Generation of ROS Autophagic cell death ER stress microRNA Oncosuppressive Apoptotic cell death Mitochondrial oxidation	Induction of apoptosis	[71,72]
Triptolide	107985	*Tripterygium wilfordii* Hook. f. Celastraceae family	Anticancer, autoimmune, inflammatory, and anti-neurodegenerative	MMPs caveolin-1 CD147 AR signaling and TFIIH RNA polymerase II	Apoptotic cell death	Induction of apoptosis	[73,74]

**Table 2:** Collection of marine bioactive compounds and their therapeutic applications in the prevention and management of prostate cancer.

Bioactive Compound	PubChem CID	Organism Name	Active Marine Compound Concentration Used	Signaling Pathway Downregulation Against Prostate Cancer	Signaling Pathway Upregulation Against Prostate Cancer	Mechanism of Action Against Prostate Cancer	References
Kahalalide F	9898671	*Elysia rufescens* (Pease, 1871) (mollusk)	PC3 cell line with an IC_50_ value of 0.07 µM was used. DU145 cell line with an IC_50_ of 0.28 µM was used.	Decrease in the level of prostate-specific antigen	Apoptosis cell death	Induction of oncosis, which refers to the process of cell death caused by a specific stimulus; potent cytotoxic activity	[181,182]
Chromopeptide A	71300966	*Chromobacterium species* (Bacteria)	Three cell lines, PC3, DU145, and LNCaP, were used; the IC_50_ values were 2.43 ± 0.02, 2.08 ± 0.16, and 1.75 ± 0.06 nmol/L, respectively.	Inhibiting cdc2 and cdc25C	Apoptotic cell death Upregulation of caspase-3	Induction of cell cycle arrest; induction of apoptosis	[183]
Mansouramycin C	44614386	*Streptomyces* sp. (Bacteria)	DU145 cell line was used, and the IC_50_ values ranged from 0.24 to 1.11 µM.	Inhibition of topoisomerase II	Apoptosis cell death ROS generation	Induction of apoptosis; inhibition of DNA replication	[182]
Demethoxy- fumitremorgin C	10337896	*Aspergillus fumigatus* Fresen. (Marine fungus)	PC-3 cells exhibited a 50% reduction in activity when exposed to a concentration of 100 μM.	Regulation of p53/p21-dependent cyclin-Cdk complexes	Apoptosis cell death	Induction of apoptosis; cell cycle arrest	[184,185]
Aspochalasins	20839478	*Ligia oceanica* (Linnaeus, 1767) (Marine fungus)	PC-3 cell growth was inhibited by an IC_50_ of 30.4 μM.	Downregulation of TNF- alpha	Apoptosis cell death	Inhibition of melanogenesis; induction of apoptosis	[186]
Rhizochalin	44445587	*Rhizochalina incrustata* (Dendy, 1922) (Marine Sponge)	The cytotoxicity of the compound was studied in several cell lines. The IC_50_ values were found to be 16.55 μM, 10.75 μM, 7.88 μM, and 7.37 μM in PC-3 cells, DU-145 cells, LNCaP cells, and another cell line, respectively.	Reduced cell migration, inhibition of cell proliferation	Apoptosis cell death	Inhibition of autophagy; arrest in the G2/M phase of the cell cycle; induction of apoptosis	[187,188]
Rhizochalinin	101464218	*Rhizochalina incrustata* (Dendy, 1922) (Marine Sponge) semi-synthetic compound derived from Rhizochalin	The cytotoxicity of the compound was measured in various cell lines. The IC_50_ values were found to be 1.14 μM in PC-3 cells, 1.05 μM in DU-145 cells, 1.69 μM in LNCaP cells, 0.87 μM in 22Rv1 cells, and 0.42 μM in VCaP cells.	Inhibition of cell migration, downregulation of AR-V7, PSA, and IGF-1 expression	Activation of ERK1/2 signaling pathway Apoptosis cell death	Inhibition of cell migration; inhibition of voltage-gated potassium channels	[187]
Halichondramide	6443267	*Chondrosia corticata* (Thiele, 1900) (Marine Sponge)	The cytotoxicity of the compound was measured in PC-3 cells, with an IC_50_ value of 0.81 μM.	Downregulation of mRNA expressions of MMP2 and MMP9, suppression of the expression of PRL-3	N-cadherin-associated upregulation of E-cadherin expression	Antimetastatic and antiproliferative activities	[189]
Latrunculin A	445420	*Negombata magnifica* (Keller, 1889) (Marine Sponge)	A suppression of the process of invasion was seen, with a 23% reduction observed at a concentration of 100 nM in PC-3 cells.	Reduced proliferation	Apoptosis cell death	Induction of apoptosis; cell cycle arrest; inhibiting prostate cancer from migrating PC3M-CT+ (calcitonin-overexpressing)	[182]
Spongistatin 1	9898465	*Spirastrella spinispirulifera* (Carter, 1879) (Marine Sponge)	An antiproliferative effect of LNCaP cells by 50% was seen at a concentration of 500 pmol.	Inhibition of proliferation	Apotosis cell death Upregulation of BIM and BCL-2 expression	Induction of apoptosis and caspase-independent cell death in DU-145 cell line; disruption of microtubules; cell cycle arrest	[190]
Niphatenone B	56946740	*Niphates digitalis* (Lamarck, 1814) (Marine Sponge)	The proliferation of LNCaP cells was significantly inhibited by 90% at a concentration of 250 μM.	Suppression of proliferation	Apoptosis cell death	Blocking of AR transcriptional activity; induction of apotosis	[191]
Agelasine B	6439899	*Agelas clathrodes* (Schmidt, 1870) (Marine Sponge)	The cytotoxicity of the substance was measured using DU-145 cells, with an IC_50_ value of 0.04 μg/mL.	Inhibition of cell proliferation, inhibition of angiogenesis	Apotosis cell death	Inhibition of the viability of PC-3 cells; reduction in Ca^2+^ concentration, triggering the fragmentation of DNA; induction of apoptosis	[192]
Lagunamide C	56839938	*Lyngbya majuscula* Harvey ex Gomont, 1892 (Marine Algae)	The cytotoxicity of the compound was measured in PC-3 cells, with an IC_50_ value of 2.6 nM.	Inhibition of cell proliferation	Apoptosis cell death	Induction of apoptosis; cell cycle arrest, specifically in the G2/M phase; inhibition of angiogenesis	[193]
Cryptophycin 52	9939639	*Nostoc* spp. (Marine Algae)	Apoptosis occurred in 40% of LNCaP cells exposed to 250 μg/mL.	Inhibition of cell migration	Upregulation of p53, bax, and p21 expression Apoptosis cell death	Cell cycle arrest; induction of apoptosis; inhibition of angiogenesis	[194]
C-phycocyanin (C-PC)	488446148	*Limnothrix* spp. (Marine Algae)	Proliferation is inhibited by 30% at 500 µg/mL in LNCaP cells.	PI3K/Akt and MAPK signaling	Apoptosis cell death Upregulation of caspases 9 and 3 activities	Induction of apoptosis; inhibition of angiogenesis	[195]
Lejimalide B	44423957	*Eudistoma* cf. *rigida* (Tokioka, 1955) (Marine Algae)	Lejimalide B dose of 30 nM resulted in the termination of the cell cycle during the G0/G1 phase.	Blocked cell cycle during the G0/G1 phase	Apoptosis cell death	Induction of apoptosis; cell cycle arrest	[196]
Astaxanthin	5281224	*Haematococcus pluvialis* (Flotow) Rabenhorst (Marine Algae)	A suppression of cell growth (38% reduction at a concentration of 0.01 μg/mL) was recorded in LNCaP cells.	Blocked NF-kB signaling pathway	Apoptosis cell death	Cell cycle arrest; induction of apoptosis	[197]
Fucoidan	129532628	*Undaria pinnatifida* (Harvey) Suringar, 1873 and *Cladosiphon novaecaledoniae* Kylin, 1940 (Marine Diatoms)	The percentage of apoptosis in PC3 cells was 15.2%, 29.8%, 39.3%, and 45.1% at concentrations of 10, 50, 100, and 200 μg/mL.	Inactivation of PI3K/Akt pathways; reduced expression of E2F-1 proteins; reduced VEGF expression; deactivation of ERK1/2 MAPK and p38 MAPK	Apoptosis cell death	Inhibition of angiogenesis; indcution of apoptosis	[198]
Fucoxanthin	5281239	*Sargassum* spp. (Marine Diatoms)	The proliferation of LNCaP cells was inhibited by 50% at 2.5 μM.	Reduced VEGF and MMP expression	Apoptosis cell death	Suppressive effects on growth demonstrated by stimulation of GADD45A; cell cycle arrest at G1	[199]
Frondoside A	44448161	*Cucumaria frondosa* (Gunnerus, 1767) (Holothurians)	Cell cycle arrest occurred in the G2/M phase in PC-3 cells when a concentration of 0.5 μM was reached.	Inhibition of proliferation	Apoptosis cell death	Terminating cell cycle and blocking autophagy; inhibition of the immune response; induction of apoptosis; inhibition of angiogenesis	[200,201]

**Table 3:** Clinical insights into plant- and marine-derived bioactive compounds in prostate cancer management.

Bioactive Compound	Source	Mechanism of Action	Findings in Clinical Trials	References
Curcumin	Turmeric (*Curcuma longa* L.)	Induces apoptosis, inhibits NF-κB and androgen receptor (AR) signaling	Improved PSA levels and reduced tumor progression in combination with standard therapies	[266]
Epigallocatechin-3-gallate (EGCG)	Green tea (*Camellia sinensis* (L.) Kuntze)	Modulates AR signaling, induces apoptosis, inhibits angiogenesis	Reduced PSA progression in patients with early-stage prostate cancer	[267,268]
Lycopene	Tetraterpene carotenoid family, found in tomato	AR signaling, NF-κB pathway IGF-1 level JNK pathway and Akt pathway	Reduced PSA progression in patients with early-stage prostate cancer	[269,270]
Sulforaphane	*Brassica oleracea* L.	PI3K/Akt and NF-kB axes Lipogenesis and lipid-dependent metabolism Glycolysis Penthose Phosphate shunt Metastasis promoter AR function PCa cell stemness	Reduction in PSA levels achieved in patients receiving sulforaphane treatment as a measure of anti-tumor activity in men with recurrent prostate cancer	[271]
White Button Mushroom extract	White button mushroom (*Agaricus bisporus* (L.) Imbach)	Disrupts AR signaling, reduces PSA expression and cell proliferation	Lowered PSA levels and suppressed tumor growth in prostate cancer patients	[272]

## Data Availability

The datasets generated and/or analyzed during the current study are available from the corresponding author on reasonable request.
